# Ethics in HTA: Examining the "Need for Expansion"

**DOI:** 10.15171/ijhpm.2017.43

**Published:** 2017-04-05

**Authors:** Payam Abrishami, Wija Oortwijn, Bjørn Hofmann

**Affiliations:** ^1^ National Health Care Institute, Diemen, The Netherlands.; ^2^ Department of Health, Ethics and Society, School CAPHRI, Maastricht University, Maastricht, The Netherlands.; ^3^ Ecorys Nederland, Sector Health, Rotterdam, The Netherlands.; ^4^ The Norwegian University of Science and Technology (NTNU), Gjøvik, Norway.; ^5^ University of Oslo, Oslo, Norway.

**Keywords:** Health Technology Assessment (HTA), Ethical Analysis, Organizational Decision-Making, Resource Allocation

## Abstract

The article by Daniels and colleagues on expanding the scope of health technology assessment (HTA) to embrace ethical analysis has received endorsement and criticism from commentators in this journal. Referring to this debate, we examine in this article the extent and locus of ethical analysis in HTA processes. An expansion/no-expansion framing of HTA is, in our view, not very fruitful. We argue that meaningfulness and relevance to the needs of the population are what should determine the extent of ethics in HTA. Once ‘relevance’ is the guiding principle, engaging in ethical analysis becomes inevitable as values are all over the place in HTA, also in how assessors frame research questions. We also challenge dividing the locus of ethical analysis into assessment and appraisal as this would detach HTA from its purpose, ie, supporting legitimate decision-making. Ethical analysis should therefore be considered integral to the HTA process.

An editorial by Daniels and colleagues entitled, “*Expanded HTA: Enhancing Fairness and Legitimacy*,”^1^ has set forth a debate accompanied by a number of successive commentaries in this journal.^2-6^ The debate addresses a core issue regarding the role of health technology assessment (HTA) for legitimising decisions on health interventions. Central to the debate is a call to broaden the scope of HTA to embrace social and ethical issues such as equity and distributional impacts. The discussion also touches a longstanding, still unfinished, debate on the locus of such analyses within HTA processes: whether ethical appraisal of a health intervention is – in terms of content – separate from its technical assessment or interwoven with it. In this article we contribute to this debate with a view to examining the extent and locus of ethical inquiry in HTA.

## To Expand or not to Expand?

As we see it, “expansion” involves a problematic framing for the scope of HTA. Expansion entails surpassing a boundary. Likewise, a ‘no-extension’ argument, as Culyer makes,^2^ involves the underlying assumption that we are deviating from a pre-existing assessment framework already demarcated by a specific discipline (medical science, epidemiology, health economics, or otherwise) and generally agreed upon within the HTA community. This view inevitably demands identifying where the boundary of expansion lies. For instance, in disagreeing with the suggestion of Daniels and colleagues to consider “matters other than safety and cost-effectiveness” in HTA, Culyer draws a line of ‘unnecessarily’ expanding HTA.

We reject a by-exclusion framing of HTA arisen from such an expansion/no-expansion argument, be it per domain or discipline, by calculation or deliberation, academic or non-academic, or otherwise. In our opinion, ‘meaningfulness and relevance’ to the needs of the population must be the prime criteria for determining the extent of HTA and for ‘sufficiency’^5^ of analyses. As a tool to inform decision-making regarding health interventions, HTA must remain user-centred in the same fashion that airlines services must be tailored to the needs of passengers or health services to those of patients. The extent of an assessment (its evaluative scope) should, in turn, be fit for the purpose of ‘legitimising decisions,’^4^ from both a practical and an epistemological point of view. A fit-for-purpose HTA is neither reductionistic nor unnecessarily exhaustive in terms of types of disciplinary perspectives, stakeholders involved, and the application of algorithmic calculations or deliberative processes. In a similar vein, an extensive elaboration of general ethical principles may in certain circumstances be rendered unnecessary, as equally may sophisticated modelling techniques. Notwithstanding, the health intervention in question should determine the content of HTA.^2^

## To Expand or to Integrate?

Once ‘relevance’ is the guiding principle, it must be justified with adequate reasoning. Engaging in ethical analysis then becomes inevitable, thereby, *integral* to HTA processes. For example, HTA influences how pooled, but scarce resources eventually address the needs of the population; and the selection and/or exclusion of issues to address in HTA reports has normative bearings.^7^ If no significant ethical issue is conceivable for the health intervention at hand, an elaborate ethical analysis may not be necessary (eg, in a case of a new me-too anti-cholesterol drug). Note: this is an if-clause. To ascertain the conditions of this ‘if,’ the assessor will inevitably have to examine budget, distributional, and financial protection impact. Nevertheless, several surveys and reviews show that HTA reports seldom explicitly address ethical issues.^1,8-12^ Some commentators in this journal reiterate this and endorse the integration of ethical analysis, including equity and fairness considerations into HTA. Culyer, however, argues that not all HTA reports need ethics; and that unveiling all specific ethical judgements would, in fact in itself, be unethical.^2^ We agree with Culyer and others who assert that not all ethical aspects of all health interventions have to be addressed for every HTA.^13^ Ethical analysis is, again, better conceived in accordance to the ‘relevance’ argument. This would also prevent inconsistency, eg, claiming that the job of assessors is to “populate the HTA process with ideas and evidence,” while regarding the equity and distributional impacts of the health intervention as “excessive.”^2^

Moreover, the fact that ethics is already embedded in HTA processes – notably in terms of minimizing opportunity costs – does not guarantee adequate ‘ethical reasoning’ or ‘social learning’ (eg, regarding societal values beyond health gains that cannot be easily quantified).^3,10^ Nor does this preclude one from examining all the value judgements underlying calculative assessments, including the relevance of cut-off points, outcome measures, time frame, indirect medical costs or costs outside the health care system, cost-effectiveness thresholds, and trade-offs between advantages and disadvantages of different measurement methodologies, to name but a few.^7,14^ These choices and assumptions are often not made explicit in an HTA report. They, however, could nontrivially influence patterns of utilization and eventually resource (re-)distribution. The devil would then be in these details, challenging Culyer’s idea of the intrinsic innocence of the tool: “Frequently what is wrong is not the tool but its users or the environment….” Are not you then engaging in an ethical analysis with regard to justifying the scope and tool of the assessment? This is indeed separate from the fact that equity considerations in resource distribution are partly captured by “a *correctly-perceived* idea of cost-effectiveness analysis in health care.”^2^

## Division of Labour Between the Two Cultures: Can You Keep Your Hands Off?

Ambivalence exists about where ethical issues should be handled: is it the task of decision-makers or do we want most of them integrated in HTA analyses? The same commentator, who argues that HTA frameworks should allow decision-makers to consider “all relevant, quantitatively and ethically significant issues,” also asserts that “[i]t is not necessary – indeed it is unethical – to prescribe all the specific ethical judgments that may have to be made. That is not the job of analysts but of decision-makers and their advisers.”^2^ In saying that HTA should not become prescriptive, Culyer notes that (*a*) scientific evidence should not be the ‘sole basis’ for making decisions and (*b*) responsibility and discretion rest on the decision-maker rather than the assessor. We agree and re-emphasise these points. However, we strongly doubt whether aiming at minimal ‘meddling’ with the job of decision-makers renders the assessments relevant or well-reasoned. The calculative assessments may either lose practical relevance or they may be regarded by the decision-maker and the public as prescriptive, not because their evaluative scope is adequate, but since they carry the connotation of being impartial or objective.^15^ Acknowledging and explicitly addressing ethical issues along with technical assessment provide the decision-maker with a more balanced/nuanced input for deciding on a certain course of action. Rather than prescriptively limiting the choices, such an ethical analysis clarifies existing choices made and the consequences thereof, even if it does not open up previously-neglected choices. It also feeds rather than discourages evidence-informed deliberative processes.^16^

It is widely acknowledged that the division between assessors and decision-makers, between fact (assessment) and value (appraisal), and between technocratic and political legitimisation has made HTA processes fall short to properly address ethical issues.^3,4,10,12,16,17^ Such division could be incongruent with the relevance argument. To those who promote this division, it should not matter if the conclusions of the assessments – having been pushed in an algorithmic direction^2,18^ – are ignored. It would, then, be defeating the purpose to expect legitimisation and transparency of decisions by means of HTA, while at the same time believing that assessors are only there to deliver evidence at the door. This leaves a lot of room for a decision-maker’s *ad hoc* personal feelings/expedience/interest, raising the question of why HTA is needed in the first place. Making decisions to optimize value^10^ is indeed the authority and responsibility of decision-makers, its ‘relevance and reasonableness’ however, relies on the work of assessors. The choice is, as Daniels and van der Wilt put it, “between HTA remaining a source of incomplete advice…, thus risking an important kind of marginalization, and HTA…to provide as complete an assessment of a technology as possible” (p. 12).^10^ You may have to choose: keeping your hands off or having an impact, because if HTA wants to have an impact on decisions, its hands may very well become openly involved.

## Conclusion

We argue that the relevance to the decision at hand is what should determine the content of HTA. Ethical underpinnings of cost-effectiveness analyses do not, in themselves, assure adequate ethical reasonableness in an HTA. Ethical analysis is integral to the whole HTA process as it contributes to how HTA is defined, interpreted, and acted upon. It includes equity and distributional considerations but also all value judgments inherently involved in assessments. To examine the extent and the role of ethical analysis in HTA, we may need to make up our mind: becoming detached from or catering to the needs of the population.

## Ethical issues

Not applicable.

## Competing interests

Authors declare that they have no competing interests.

## Authors’ contributions

PA drafted the manuscript. All authors then contributed to the concept and writing of the article.

## Authors’ affiliations

^1^National Health Care Institute, Diemen, The Netherlands. ^2^Department of Health, Ethics and Society, School CAPHRI, Maastricht University, Maastricht, The Netherlands. ^3^Ecorys Nederland, Sector Health, Rotterdam, The Netherlands. ^4^The Norwegian University of Science and Technology (NTNU), Gjøvik, Norway. ^5^University of Oslo, Oslo, Norway.
